# Effects of Sodium Intake on Health and Performance in Endurance and Ultra-Endurance Sports

**DOI:** 10.3390/ijerph19063651

**Published:** 2022-03-19

**Authors:** Eleftherios Veniamakis, Georgios Kaplanis, Panagiotis Voulgaris, Pantelis T. Nikolaidis

**Affiliations:** 1 Department of Nutrition and Dietetics, Hellenic Mediterranean University, 72300 Sitia, Greece; veniamakise@gmail.com (E.V.); kaplanisgiwrgos1@gmail.com (G.K.); voulgaris.panagiotis7@gmail.com (P.V.); 2 School of Health and Caring Sciences, University of West Attica, 12243 Athens, Greece

**Keywords:** sodium, endurance sports, ultra-endurance sports, hyponatraemia, muscle cramps, hydration

## Abstract

The majority of reviews on sports nutrition issues focus on macronutrients, often omitting or paying less attention to substances such as sodium. Through the literature, it is clear that there are no reviews that focus entirely on the effects of sodium and in particular on endurance sports. Sodium intake, both at high and low doses, has been found to be associated with health and performance issues in athletes. Besides, there have been theories that an electrolyte imbalance, specifically sodium, contributes to the development of muscle cramps (EAMC) and hyponatremia (EAH). For this reason, it is necessary to create this systematic review, in order to report extensively on the role of sodium consumption in the population and more specifically in endurance and ultra-endurance athletes, the relationship between the amount consumed and the occurrence of pathological disorders, the usefulness of simultaneous hydration and whether a disturbance of this substance leads to EAH and EAMC. As a method of data collection, this study focused on exploring literature from 1900–2021. The search was conducted through the research engines PubMed and Scopus. In order to reduce the health and performance effects in endurance athletes, simultaneous emphasis should be placed on both sodium and fluid intake.

## 1. Introduction

Metals are a group of minerals that cannot be produced by the body. Sodium, being an inorganic element, is an essential component in human nutrition. As such, excessive or very low intake of this ingredient can have adverse effects on the body. So, attention should be paid to this element as well. Most reviews on sports nutrition tend to praise protein (PRO), carbohydrates (CHO) and fat (FAT) without paying much attention to sodium [1,2,3]. The loss of body fluids during sport or exercise is largely due to sweating [4]. Thus, replacement of Na+ loss in sweat is recommended when the duration of exercise is longer than 2 h, when the climate is hot or during intense Na+ loss in sweat (e.g., >3–4 g Na+) [5,6]. In endurance sports, the duration of a race is longer than 5 min [7], while in ultra-endurance sports, the duration of a race is longer than 6 h [2]. So, it is important to fully replace the losses in fluids and Na+ to restore e-hydration. There are also theories [8,9] suggesting a positive association of sodium with muscle cramps and the occurrence of hyponatremia, while the causes are attributed to the long-duration intensity of exercise, which leads to muscle fatigue, and excessive fluid consumption, mainly pure water, respectively [10,11]. Thus, the following questions arise: (a) What should be the amount of sodium in the population? (b) Do they differ from that of athletes? (c) Is there any evidence that finally confirms the link between sodium and muscle cramps and hyponatremia? (d) Can sodium eliminate the occurrence of hyponatremia? (e) What are the hydration recommendations in endurance sports? The aim of this study is to collect research and literature from 1900 to 2021 in order to clarify the causes of muscle cramps, to record studies that clarify the role of sodium in hyponatremia. Hydration recommendations will also be made before, during and after exercise, with an emphasis on both the importance of fluid consumption and the importance of insufficient hydration leading to either over-hydration or dehydration. Thus, 130 bibliographies were used to complete this work.

## 2. Importance of Sodium and Ideal Composition

Sodium chloride (common salt) is an anionic compound with an extracellular fluid concentration adjusted to about 135–145 mmol/L [12]. More specifically, sodium is the main cation in extracellular fluid [13] with a multitude of benefits for both the general population and the sporting world, such as contributing to the release of digestive secretions and controlling the absorption of certain nutrients (amino acids, glucose, galactose and water) [12,14]. In addition, it ensures sufficient blood volume, blood pressure and, ultimately, organ perfusion [15]. In addition to its importance in terms of regulating water and fluid balance [16], it is vital for the stimulation of muscle and nerve cells and is also involved in the control of the acid–base balance [17]. In the sport section, sodium helps to maintain serum electrolyte concentrations resulting in a balance of intravascular osmotic pressure and plasma volume [18]. It increases the thirst stimulus and reduces the amount of urine produced [19], effects that ultimately reduce physical fatigue and medical problems associated with these homeostatic imbalances in endurance sports [20]. However, attention should be paid to excess sodium, which contributes to high blood pressure and damage to certain organs such as the heart, kidneys and bones [21]. In contrast, a low intake has been associated with an increased risk of cardiovascular events and death, independent of blood pressure levels [22,23].

## 3. Effects of Sodium Consumption

### 3.1. Very Low Sodium Consumption

There are studies [23,24] that show that even low sodium intake may not always be beneficial for the treatment of cardiovascular disease. Low sodium intake has been associated with an increased risk of cardiovascular events and death, independent of blood pressure levels [22,24]. In the study by Mente and colleagues, it was found that urinary sodium excretion of less than 3 g per day did not further reduce systolic blood pressure but actually tended to increase diastolic blood pressure in people with or without hypertension [25]. In both normotensive and hypertensive individuals, low sodium intake can cause insulin resistance and an increase in plasma or serum levels of renin, aldosterone, epinephrine and norepinephrine [22,26]. Subsequently, in the reviews by Yin et al. [27] and Van Horn et al. [28], it appears that sodium consumption of 0.5–1.0 g per day is attributed as an optimal physiological intake, in contrast to those of Mente et al. [29] and Mente et al. [25] where concerns have been raised about potential negative health effects due to a reduction in intake below the average global consumption level. These concerns mainly come from prospective observational studies [25,30], some of which report associations between sodium intake and cardiovascular disease [31,32]. Individuals reporting low levels of sodium intake are often patients with a history of disease who have been advised to reduce sodium. Among these individuals, there may be an increased risk resulting from concomitant disease leading to adverse cardiovascular outcomes rather than low sodium intake [25,29].

### 3.2. Very High Sodium Consumption

Increased sodium intake leads to an increase in intra-glomerular pressure, which can cause or exacerbate chronic kidney damage and increase the risk of progressive kidney disease [33]. Subsequently, the mechanisms by which a high-salt diet increases the risk of gastric cancer in humans are poorly understood [34]. One speculation is that foods such as processed meat, cured meat and dried fish exposed to salt are high in nitrite compounds, which may be involved in gastric carcinogenesis [35]. Still, studies [36,37] on the association of sodium with osteoporosis suggest that increased sodium intake is a risk factor for the development of the disease (Table 1). Specifically, in postmenopausal women in Korea [37,38], it was shown that high sodium intake (>2000 mg) leads to increased urinary excretion (>2 g/day), which leads to hypercalciuria, thus increasing the risk for osteoporosis [39]. Finally, excessive salt intake by consumers has been associated with the development of hypertension [40,41] and, consequently, with a higher risk of cardiovascular disease, particularly for hypertensives and the elderly [42]. Reducing sodium intake is associated with a reduction in systolic and diastolic blood pressure, particularly in hypertensive and normotensive individuals [12,43]. 

**Figure 1:**
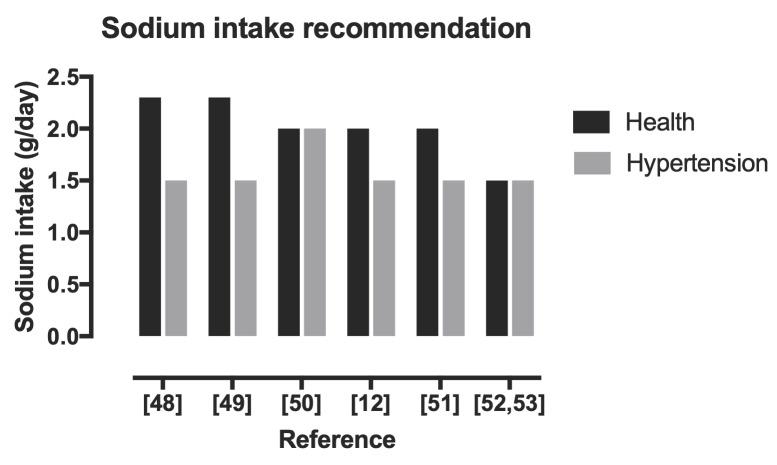
Recommendations of sodium intake [12,48,49,50,51,52,53].

Although it is possible that both low and high levels of sodium intake are harmful, the mechanism of these effects is uncertain [23]. What does seem certain, however, is that after a retrospective search during the 2010–2019 period, the ideal amount of sodium was 1.5 g/day (Figure 1). It appears that, over time, the recommendations for both the healthy population and hypertensive patients do not make much difference.

## 4. Sodium in Sport

Turning to the main issue, despite the positive effects of sodium consumption, such as maintaining aldosterone and vasopressin production [54], increasing thirst stimulation and decreasing urine production [55], enhancing electrolyte balance and stimulating water retention in the body, resulting in a reduction of physical fatigue in endurance sports [56], it has been implicated by previous theories [8,9] that it contributes, positively, to the occurrence of muscle cramps and hyponatremia during exercise.

### 4.1. Exercise-Associated Muscle Cramps 

Exercise-associated muscle cramps (EAMC) are defined as painful, spasmodic and involuntary contractions of skeletal muscles during or immediately after physical activity [10,57]. The prevalence of EAMC in different sports varies, as shown in Table 2. The basic etiological evidence in the literature by Schwellnus et al. [8] is that electrolyte depletion through excessive sodium loss in sweat along with dehydration causes this condition. These causes do not offer plausible pathophysiological mechanisms with supporting scientific evidence that could adequately explain their clinical presentation and management. Thus, studies were conducted [10,57] whose results showed that dehydration and sodium depletion do not appear to be associated with muscle cramps.

### 4.2. Exercise-Associated Hyponatremia

Exercise-associated hyponatremia occurs when plasma sodium concentration is <135 mmol/L or there is a decrease in serum sodium of 7–10% [63,64]. This can occur during or after prolonged exercise for 4 to 6 h or more [65] and can be detected up to 24 h after the end of exercise [63,66,67,68]. It is a disorder that has been widely described in marathon runners [67] but also in athletes and in other endurance and super-endurance events. Ultra-endurance athletes competing in events longer than 24 h, such as participants in the Ultraman, Titan Desert or Sables Marathon, are at a higher risk of developing Exercise-Associated Hyponatremia (EAH) compared to participants in shorter endurance events such as the marathon (Table 3.) [67,68,69]. However, the environment also contributes as a factor to hyponatremia [67,70]. The main causes of this condition appear to be excessive fluid consumption during exercise, increased sodium loss in sweat and loss of normal Antidiuretic hormone (ADH) suppression, called the syndrome of inappropriate ADH secretion (SIADH) [71]. In a more recent study, that of Buck and colleagues [72], EAH was found to be due to both increased consumption of hypotonic fluids [73] and inappropriate water retention [74] The contribution of sodium loss from excessive sweat is controversial as sweat loss varies greatly between individual athletes but typically ranges between 15 and 65 mEq/L and sweat volume ranges from 250 mL/h to >2 L/h. Therefore, sodium lost from sweat is not thought to be responsible in itself for the development of EAH, but rather an additive effect along with hyperhydration. The symptoms of hyponatremia as seen in Table 4 vary [63,72,75,76,77,78,79]. Because some symptoms of hyponatremia can be identified with other conditions, the clinical sign that differentiates hyponatremia from other conditions that result in collapse is vomiting. Vomiting may be a reflex action in response to the increasing distension of large and unnecessary amounts of fluid within the gastrointestinal tract or may be caused by the central nervous system [80]. However, the presence of cognitive impairment, coma, seizures or respiratory distress suggests exercise-associated hyponatremic encephalopathy and should be recognized immediately [81], as it has been confirmed as a cause of at least fourteen deaths [77].

#### Prevention and Treatment

EAH can be prevented by avoiding over-hydration, ensuring adequate oral sodium intake and training athletes, focusing on sweat rate and sweat sodium content, exercise intensity and environmental conditions [11,72]. Estimates of individual athlete replacement needs can also be used by monitoring weight changes during training activities, although this may not be practical. Forced hydration, particularly in large quantities, should be discouraged [75,97]. Before treatment, it is very important to differentiate EAH from other exercise-related diseases such as heat exhaustion, heat stroke and exercise-induced collapse associated with sickle cell anemia, as their treatments are often contradictory [72]. If a patient has no neurological symptoms, EAH is considered mild and oral fluid restriction is required (Table 5). In studies by Siegel [86] and Bridges [98], oral HTS (hypertonic saline) reduces symptomatology from EAH faster than an IV bolus (intravenous administration). Unfortunately, oral hypertonic fluids may not be palatable [99], which limits their usefulness. Therefore, knowing that the main cause of EAH is the habit of excessive fluid consumption during a race combined with inadequate sodium intake [90], it is noteworthy to provide a full description of both hydration and incorrect fluid intake practices, as well as fluid and sodium quantities.

## 5. Sodium and Hydration

There are some studies [102,103] that have been dedicated to determining the benefits of salt intake on endurance performance. Most of them report improved physical performance, an attenuated decrease in serum sodium concentration and enlarged plasma volume during endurance activities. It is worth noting that laboratory studies have always accompanied salt supplementation during exercise with a liquid ingestion pattern that matches sweat losses, which probably facilitated the occurrence of these benefits. In general, the water needs of athletes tend to vary according to individual characteristics and the type or intensity of exercise in which they participate, making individualized fluid replacement strategies necessary [102,103]. This should result in both preventing adverse effects and improving the performance of athletes [104] and maintaining proper hydration during exercise [105]. Athletes participating in endurance and ultra-endurance races should be aware that both prior acclimatization to race weather conditions and adequate fluid–electrolyte balance reduce the risk of dehydration and thus the risk of EAH [106]. The loss of body fluids during sport or exercise is largely due to sweating [107].

It is primarily a function of heat production by metabolism, but can be modified by the environment, clothing, acclimatization, hydration status, the size and composition of the athlete’s body and the degree of training [108,109,110]. However, this heat dissipation is accompanied by typical fluid losses of 0.5–1.9 L/h according to Baker and colleagues [110]. Thus, the goals are to improve athlete performance, maintain proper hydration during exercise, prevent adverse effects (dehydration-hyperhydration) and avoid losses greater than 2–3% of body mass during exercise [104,105]. Dehydration causes a loss of intracellular and extracellular (plasma and interstitial) fluid in proportion to water loss, compromising cardiovascular function, reducing muscle blood flow and cardiac output [111]. Typically, sweat is hypotonic (i.e., lower concentration of electrolytes) compared to plasma [112]. Therefore, exercise-related sweat losses lead to a decrease in plasma volume and an increase in plasma electrolyte concentration (primarily sodium), known as hypertonic hypovolemia [112]. Low blood volume, due to dehydration, also prevents the transport of oxygen and glucose to muscle cells [111]. Dehydration of 2% of the body weight, which generally occurs during exercise lasting more than 90 min, appears to significantly reduce endurance performance in 20–21 °C environments [113]. Further, weight loss of more than 4% of the body weight during exercise can lead to heat illness, heat exhaustion, heat stroke and possibly death [114].

Athletes may drink large amounts of fluids in the hours before competition, often in combination with an osmotic agent such as glycerol or sodium, in order to temporarily increase total body water to compensate for sweat losses and delay the progression of absolute hypohydration [115]. This practice has been identified as a result of incorrect guidelines for fluid intake in sport, and when carried out in extreme situations, can lead to serious consequences associated with hyponatremia [77]. Therefore, avoiding over-hydration or under-hydration is recommended for both health and performance in ultramarathon running. In some endurance and ultra-endurance races, it has affected up to 30% of participants [116]. However, various dosages have been suggested for before, during and after exercise as shown in Table 6, Table 7 and Table 8. These dosages are chosen according to the athlete’s tolerance to fluid volume.

## 6. Sources and Dosages of Sodium in Endurance–Ultra-Endurance Sports

In the review by Grozenski and Kiel [125], the consumption of drinks with 20 to 50 mEq-L sodium or small amounts of salted snacks helps to stimulate thirst, reabsorption of fluids and, by extension, support osmotic balance during endurance events. Additional salting of everyday foods is an inexpensive and effective method of increasing sodium intake (pickles, tomato juice, canned soups, baked beans and pizza) [56]. 

Furthermore, adding 3.2 gr (0.5 teaspoons) of table salt to every 960 mL (32 fl oz) of a sports drink will further increase sodium concentration without negatively affecting taste or absorption [56]. Additionally, Tiller’s [121] study argues that in order to reduce the risk of hyponatremia during long-duration exercise, runners should consume sodium at concentrations of 500–700 mg-L of fluid [118]. Slightly higher amounts of sodium (and other electrolytes) will be required under conditions of heat (e.g., >25 °C/77 °F) and/or humidity (e.g., >60%). When the sweat rate is elevated in such conditions, runners should aim for 300–600 mg-h-sodium (1000–2000 mg NaCl). If consumed in liquids, sodium concentrations greater than 1000 mg-L (50 mmol-L) should be avoided as this may reduce the palatability of the drink [4]. The amounts ingested should also be offset against the sodium consumed from salt-containing foods, although it should be noted that it is unlikely that the recommended sodium intake rate from food alone will be achieved. The Academy of Nutrition and Dietetics (AND), Dietitians of Canada (DC) and The American College of Sports Medicine (ACSM) recommend sodium intake during exercise in athletes with high sweat rates (>1.2 L/h), subjective “salty sweating” and prolonged exercise >2 h [119]. Although highly variable, average sweat rates range from 0.3 to 2.4 L/h, and the average sweat sodium content is 1 gr/L (50 mmol/L) [119]. A sports drink containing sodium in the range of 10–30 mmol/L (230–690 mg/L) results in optimal absorption and prevention of hyponatremia [126], a concentration found in typical commercial sports drinks. ACSM recommendations for sodium intake are 300–600 mg/h (1.7–2.9 g salt) during prolonged exercise [120,126]. However, as shown in the previous chapters, sodium intake can be positively associated with the onset of the above disorders (EAH, EAMC) [8,9].

## 7. Discussion

Sodium is the main cation of extracellular fluid that has many advantages [12,17], with one of its main functions being maintaining fluid balance in the body [56]. Moreover, as mentioned above, the right amount plays an important role, as both high consumption and low consumption are health risks [23]. Many organizations (such as the Institute of Medicine, World Health Organization recommend sodium intakes of up to 1.5 g/day. In contrast, in sports, amounts vary. It is well documented that the sweat rate and sweat electrolyte concentrations can vary significantly as a result of many factors, therefore individualized fluid replacement strategies are recommended [118]. Urine output after exercise decreases as the sodium concentration in the drink increases. Plain water is unlikely to be sufficient to restore fluid balance after exercise due to the subsequent reduction in sodium concentration and plasma osmolality that causes diuresis. Furthermore, sodium intake should ideally be equal to the sodium concentration lost in sweat. The sodium content of commercial sports drinks (~20–25 mmol-L, 460–575 mg-L) is lower than that normally lost in sweat [127] and should also be considered a conservative target. Regarding muscle cramps, there does not appear to be documented scientific evidence for the sodium–EAMC relationship. The most common cause of this condition is exercise at a higher relative intensity or exercise duration compared to normal training, resulting in muscle fatigue [10]. While sodium intake during a race can mitigate the drop in blood sodium concentrations, it cannot prevent EAH under conditions of excessive fluid intake [128]. Sodium intake during exercise will not prevent EAH in the presence of hyperhydration, but excessive sodium intake may actually increase the risk of EAH [129]. It is the amount of fluid, not the amount of sodium consumed, during exercise that increases final blood sodium concentrations. Sodium-containing sports drinks that are hypotonic will not prevent EAH in athletes who drink excessively during exercise [130]. Athletes should be trained to tolerate drinking larger amounts of water and ensure they consume more fluids in hotter and more humid environments. Sports nutritionists, dietitians and sports coaches can play an important role in educating athletes and coaches on proper hydration methods and overseeing fluid intake during training and competition. The goal is to limit weight loss to 2% [125].

## 8. Conclusions

Sodium is an element that should not be missing from people’s diets. Thus, the ideal amount of sodium intake in the largest range of the population appeared to be in the range of 1.5 g/day. However, it is equally important for endurance athletes to consume 300–600 mg/h. It was also noted that there is no documented scientific evidence on the relationship between sodium and muscle cramps. Sodium seems to be one, but not the only, factor contributing to this situation. For hyponatremia, its intake can mitigate the drop in blood concentrations, but cannot eliminate it. Finally, attention should first be paid to the individual amount of fluids consumed and then to the amount of sodium consumed.

## Figures and Tables

**Table 1:** Relationship of clinical conditions with high sodium intake.

Clinical Condition	n	Average Age (Years)	Average Sodium Intake	Τype of Study	Correlation	References
Kidney Disease	1384	≥20	11.5 gr	Observational	Positive	[44]
Cancer	2485	18–92	9 g	Case-control	Positive	[45]
Cancer	634	40–49	12.8 g	Cross-sectional	Positive	[46]
Hypertension	3230	22–73	9.4 g	Meta-analysis	Positive	[47]
Hypertension	10,074	20–59	Serum > 100 mmol/L	Cross-sectional	Positive	[12]
Osteoporosis	537	58 ± 6	>2 g/day	Cross-sectional	Positive	[37]
Osteoporosis	102	24 ± 3.4	2.6 ± 1.1 g/day	Cross checked	Positive	[36]

**Table 2:** Prevalence of exercise-associated muscle cramps (EAMC) in athletes.

Sports	Prevalence EAMC	References
Ultra-Marathon 166 km	14%	[58]
Marathon	18%	[10]
Ironman Triathlon	23%	[59]
Ultra-Marathon 100 km	23%	[60]
Ultra-Marathon 56km	41%	[59]
Cycling	60%	[8]
American football	30–53%	[61,62]

**Table 3:** Prevalence of exercise-associated hyponatremia.

Sports	Trial	Prevalence EAH	References
Marathon	Marathon	15%	[82]
	Houston Marathon 2000	<5%	[80]
	Boston Marathon	5%	[83]
	Houston Marathon 2000–2004	>20%	[84]
	Zurich Marathon	<5%	[85]
	Boston Marathon 2001–2018	<5%	[86]
	London Marathon	Up to 22%	[87]
Ultra-Marathon	Ultra-marathon in Asia	38%	[88]
	161 km in North America	30–51%	[89] [90] [91]
Cycling	109 km	12%	[92]
	210–250 km	4.5% (4 to 90 persons)	[93]
Triathlon	Ironman-Triathlon	20%	[69]
	Ironman-Triathlon	1.8–28%	[94,95]
	Triple Ironman	26%	[67]

**Table 4:** Symptoms of EAH [65,69,72,75,77,81,96].

Mild	Severe	Clinical Appearance
WearinessDizziness	Mental disorder	Heat stroke
Slow urine production	Ictus, collapse	Hypoglycemia
Sickness	Oliguria	Stress-related collapse
Headache	Coma	Muscle cramps
Weakness	Death	Edema

**Table 5:** Management of EAH symptoms.

	Mild Symptoms	Severe (Neurological Symptoms)	In Encephalopathy	Bibliography
Intravenous isotonic fluids of any type or volume are not recommended	recommended	is not recommended	is not recommended	[100]
Concentrated oral sodium replacement may be given (with reservation)	recommended	is not recommended	is not recommended	[86]
Bolus 100 mL of intravenous hypertonic saline (3% sodium chloride)	is not recommended	recommended	recommended	[63] [75]
Should be treated immediately with intravenous IV bolus infusion or HTS infusion for acute reduction of swelling in the brain	is not recommended	is not recommended	recommended	[58] [101]

**Table 6:** Pre-exercise hydration dosages.

Timing	Dosage	Bibliography
Before exercise	5 to 10 mL/kg body weight	[117]
Before exercise	5–7 mL/kg 4 h before exercise and more 3–5 mL/kg, 2 h before competition	[118]
4 h before exercise	5–7 mL/kg water or sports drink	[119]
Before exercise	400–600 mL cold water or sports drink 20–30 min before exercise	[120]

**Table 7:** Fluid intake during exercise.

Sports	Timing	Dosage	Bibliography
Ultra-Marathon	During exercise or competition, each 20 min	150–250 mlliquids	[121]
Ultra-Marathon Competition	Each 1 h	300–600 mL	[70]
Marathon	Each 1 h	400–800 mL	[118]
Regardless of sport	During the exercise	450–675 mL, for every 0.5 kg of body weight lost	[122]

**Table 8:** Fluid intake after the exercise.

Sports	Timing	Dosage	Bibliography
Regardless of sport	After the exercise	1.25 to 1.5 L liquids for every 1 kg of weight loss	[117]
General for athletes in a warm climate	After the exercise	100–120% body mass losses	[123]
Regardless of sport	For fullrestoration	450–675 mL for every 0.5 kg of weight loss	[119]
General for athletes	After the exercise	Liquid with 150% or 200% of weight loss	[124]

## Data Availability

Not applicable.

## References

[1] Stellingwerff T., Maughan R.J., Burke L.M. (2011). Nutrition for power sports: Middle-distance running, track cycling, rowing, canoeing/kayaking, and swimming. J. Sports Sci..

[2] Jeukendrup A.E. (2011). Nutrition for endurance sports: Marathon, triathlon, and road cycling. J. Sports Sci..

[3] Costa R.J.S., Camões-Costa V., Snipe R.M.J., Dixon D., Russo I., Huschtscha Z. (2019). The impact of exercise-induced hypohydration on gastrointestinal integrity, function, symptoms, and systemic endotoxin and inflammatory profile. J. Appl. Physiol..

[4] Baker L.B., Ungaro C.T., Barnes K.A., Nuccio R.P., Reimel A.J., Stofan J.R. (2014). Validity and reliability of a field technique for sweat Na+ and K+ analysis during exercise in a hot-humid environment. Physiol. Rep..

[5] Coyle E.F. (2004). Fluid and fuel intake during exercise. J. Sports Sci..

[6] Shirreffs S.M., Sawka M.N. (2011). Fluid and electrolyte needs for training, competition, and recovery. J. Sports Sci..

[7] Ganio M.S., Armstrong L.E., Kavouras S.A. (2018). Hydration. Sport and Physical Activity in the Heat.

[8] Schwellnus M.P., Drew N., Collins M. (2008). Muscle cramping in athletes—Risk factors, clinical assessment, and management. Clin. Sports Med..

[9] Knechtle B., Chlíbková D., Papadopoulou S., Mantzorou M., Rosemann T., Nikolaidis P.T. (2019). Exercise-Associated Hyponatremia in Endurance and Ultra-Endurance Performance–Aspects of Sex, Race Location, Ambient Temperature, Sports Discipline, and Length of Performance: A Narrative Review. Medicina.

[10] Martínez-Navarro I., Montoya-Vieco A., Collado-Boira E., Hernando B., Panizo N., Hernando C. (2020). Muscle Cramping in the Marathon: Dehydration and Electrolyte Depletion vs. Muscle Damage. J. Strength Cond. Res..

[11] Vitale K., Getzin A. (2019). Nutrition and Supplement Update for the Endurance Athlete: Review and Recommendations. Nutrients.

[12] Rust P., Ekmekcioglu C. (2016). Impact of Salt Intake on the Pathogenesis and Treatment of Hypertension. Hypertension: From Basic Research to Clinical Practice.

[13] Drüeke T.B. (2016). Salt and health: Time to revisit the recommendations. Kidney Int..

[14] Koepsell H. (2020). Glucose transporters in the small intestine in health and disease. Pflüg. Arch. Eur. J. Physiol..

[15] Stolarz-Skrzypek K., Bednarski A., Czarnecka D., Kawecka-Jaszcz K., Staessen J.A. (2013). Sodium and Potassium and the Pathogenesis of Hypertension. Curr. Hypertens. Rep..

[16] Farquhar W.B., Edwards D.G., Jurkovitz C.T., Weintraub W.S. (2015). Dietary Sodium and Health. J. Am. Coll. Cardiol..

[17] Mohammadifard N., Gotay C., Humphries K.H., Ignaszewski A., Esmaillzadeh A., Sarrafzadegan N. (2018). Electrolyte minerals intake and cardiovascular health. Crit. Rev. Food Sci. Nutr..

[18] Del Coso J., González-Millán C., Salinero J.J., Abián-Vicén J., Areces F., Lledó M., Lara B., Gallo-Salazar C., Ruiz-Vicente D. (2015). Effects of oral salt supplementation on physical performance during a half-ironman: A randomized controlled trial. Scand. J. Med. Sci. Sports.

[19] Shirreffs S.M., Maughan R.J. (1998). Volume repletion following exercise-induced volume depletion in man: Replacement of water and sodium losses. Am. J. Physiol..

[20] Speedy D.B., Thompson J., Rodgers I., Collins M., Sharwood K. (2002). Oral salt supplementation during ultradistance exercise. Clin. J. Sport Med..

[21] Robinson A., Edwards D.G., Farquhar W.B. (2019). The Influence of Dietary Salt Beyond Blood Pressure. Curr. Hypertens. Rep..

[22] Graudal N.A., Hubeck-Graudal T., Jurgens G. (2017). Effects of low sodium diet versus high sodium diet on blood pressure, renin, aldosterone, catecholamines, cholesterol, and triglyceride. Cochrane Database Syst. Rev..

[23] Mente A., O’Donnell M., Rangarajan S., Dagenais G., Lear S., McQueen M., Diaz R., Avezum A., Lopez-Jaramillo P., Lanas F. (2016). Associations of urinary sodium excretion with cardiovascular events in individuals with and without hypertension: A pooled analysis of data from four studies. Lancet.

[24] Braam B., Huang X., Cupples W.A., Hamza S.M. (2017). Understanding the Two Faces of Low-Salt Intake. Curr. Hypertens. Rep..

[25] Mente A., O’Donnell M.J., Rangarajan S., McQueen M.J., Poirier P., Wielgosz A., Morrison H., Li W., Wang X., Di C. (2014). Association of urinary sodium and potassium excretion with blood pressure. N. Engl. J. Med..

[26] Oh H., Lee H.Y., Jun D.W., Lee S.M. (2016). Low salt diet and insulin resistance. Clin. Nutr. Res..

[27] Yin X., Tian M., Neal B. (2020). Sodium Reduction: How Big Might the Risks and Benefits Be?. Heart Lung Circ..

[28] Van Horn L. (2015). Dietary sodium and blood pressure: How low should we go?. Prog. Cardiovasc. Dis..

[29] Mente A., O’Donnell M., Rangarajan S., McQueen M., Dagenais G., Wielgosz A., Lear S., Ah S.T.L., Wei L., Diaz R. (2018). Urinary sodium excretion, blood pressure, cardiovascular disease, and mortality: A community-level prospective epidemiological cohort study. Lancet.

[30] O’Donnell M., Mente A., Rangarajan S., McQueen M.J., Wang X., Liu L., Yan H., Lee S.Y., Mony P., Devanatah A. (2014). Urinary sodium and potassium excretion, mortality, and cardiovascular events. N. Engl. J. Med..

[31] Paterna S., Fasullo S., Cannizzaro S., Vitrano G., Terrazzino G., Maringhini G., Ganci F., Scalzo S., Di Pasquale P., Parrinello G. (2011). Short-term effects of hypertonic saline solution in acute heart failure and long-term effects of a moderate sodium restriction in patients with compensated heart failure with New York heart Association Class III (Class C) (SMAC-HF study). Am. J. Med. Sci..

[32] Paterna S., Gaspare P., Fasullo S., Sarullo F., Di Pasquale P. (2008). Normal-sodium diet compared with low-sodium diet in compensated congestive heart failure: Is sodium an old enemy or a new friend?. Clin. Sci..

[33] Suzuki H., Takenaka T., Kanno Y., Ohno Y., Saruta T. (2007). Sodium and Kidney Disease. Nutrition and Kidney Disease: A New Era. Contribution to Nephrology.

[34] Loh J.T., Torres V., Cover T. (2007). Regulation of Helicobacter pylori cagA Expression in Response to Salt. Cancer Res..

[35] World Cancer Research Fund⁄American Institute for Cancer Research (2007). Food, Nutrition, Physical Activity, and the Prevention of Cancer: A Global Perspective.

[36] Bedford J.L., Barr S.I. (2011). Higher urinary sodium, a proxy for intake, is associated with increased calcium excretion and lower hip bone density in healthy young women with lower calcium intakes. Nutrients.

[37] Park S.M., Joung J.Y., Cho Y.Y., Sohn S.Y., Hur K.Y., Kim J.H., Kim S.W., Chung J.H., Lee M.K., Min Y.K. (2015). Effect of high dietary sodium on bone turnover markers and urinary calcium excretion in Korean postmenopausal women with low bone mass. Eur. J. Clin. Nutr..

[38] Park Y., Kwon S.J., Ha Y.C. (2016). Association between Urinary Sodium Excretion and Bone Health in Male and Female Adults. Ann. Nutr. Metab..

[39] Kim S.-W., Jeon J.-H., Choi Y.-K., Lee W.-K., Hwang I.-R., Kim J.-G., Lee I.-K., Park K.-G. (2015). Association of urinary sodium/creatinine ratio with bone mineral density in postmenopausal women: KNHANES 2008–2011. Endocrine.

[40] O’Donnell M., Mente A., Yusuf S. (2015). Sodium intake and cardiovascular health. Circ. Res..

[41] Whelton P.K., Appel L.J., Sacco R.L., Anderson C.A.M., Antmann E.M., Campbell N., Bunbar S.B., Frohlich E.D., Hall J.E., Jessup M. (2012). Sodium, blood pressure, and cardiovascular disease: Further evidence supporting the American Heart Association sodium reduction recommendations. Circulation.

[42] Irish Heart Foundation (2008). Salt, Blood Pressure and Heart Disease. http://www.irishheart.ie/iopen24/pub/healthpromotionreports/ihfstatement_salt.

[43] Whelton P.K., He J. (2014). Health effects of sodium and potassium in humans. Curr. Opin. Lipidol..

[44] Sugiura T., Takase H., Ohte N., Dohi Y. (2018). Dietary Salt Intake is a Significant Determinant of Impaired Kidney Function in the General Population. Kidney Blood Press. Res..

[45] Peleteiro B., Lopes C., Figueiredo C., Lunet N. (2010). Salt intake and gastric cancer risk aαccording to Helicobacter pylori infection, smoking, tumour site and histological type. Br. J. Cancer.

[46] D’Elia L., Galletti F., Strazzullo P. (2013). Dietary Salt Intake and Risk of Gastric Cancer. Cancer Treat. Res..

[47] He F.J., Li J., MacGregor G.A. (2013). Effect of longer term modest salt reduction on blood pressure: Cochrane systematic review and meta-analysis of randomised trials. BMJ.

[48] Institute of Medicine (2005). Dietary Reference Intakes for Water, Potassium, Sodium, Chloride, and Sulfate. Panel on Dietary Reference Intakes for Electrolytes, Water.

[49] US Department of Agriculture and US Department of Health and Human Services (2010). Dietary Guidelines for Amercans.

[50] WHO (2012). Guideline Sodium Intake for Adults and Children.

[51] Grillo A., Salvi L., Coruzzi P., Salvi P., Parati G. (2019). Sodium Intake and Hypertension. Nutrients.

[52] Flack J.M., Adekola B. (2019). Blood Pressure and the New ACC/AHA Hypertension Guidelines. Trends Cardiovasc. Med..

[53] Oria M., Harrison M., Stallings V.A. (2019). National Academies of Sciences, Engineering, and Medicine; Health and Medicine Division; Food and Nutrition Board; Committee to Review the Dietary Reference Intakes for Sodium and Potassium.

[54] Rehrer N.J. (2001). Fluid and electrolyte balance in the ultra-endurance sport. Sports Med..

[55] Clapp A.J., Bishop P.A., Smith J.F., Mansfield E.R. (2000). Effects of Carbohydrate-Electrolyte Content of Beverages on Voluntary Hydration in a Simulated Industrial Environment. AIHAJ Am. Ind. Hyg. Assoc..

[56] Valentine V. (2007). The importance of salt in theathlete’s diet. Curr. Sports Med. Rep..

[57] Maughan R.J., Shirreffs S.M. (2019). Muscle Cramping During Exercise: Causes, Solutions, and Questions Remaining. Sports Med..

[58] Hoffman M.D., Stuempfle K.J. (2015). Muscle Cramping During a 161-km Ultramarathon: Comparison of Characteristics of Those with and without Cramping. Sports Med. Open.

[59] Schwellnus M.P., Allie S., Derman W., Collins M. (2011). Increased running speed and pre-race muscle damage as risk factors for exercise-associated muscle cramps in a 56 km ultra-marathon: A prospective cohort study. Br. J. Sports Med..

[60] Kao W.F., Hou S.K., Chiu Y.H., Chou S.L., Kuo F.C., Wang S.H., Chen J.J. (2015). Effects of 100-km ultra marathon on acute kidney injury. Clin. J. Sport Med..

[61] Greenwood M., Kreider R.B., Greenwood L., Byars A. (2003). Cramping and injury incidence in collegiate football players are reduced by creatine supplementation. J. Athl. Train..

[62] Maddali S., Rodeo S.A., Barnes R., Warren R.F., Murrell G.A. (1998). Postexercise increase in nitric oxide in football players with muscle cramps. Am. J. Sports Med..

[63] Bennett B.L., Hew-Butler T., Rosner M.H., Myers T., Lipman G.S. (2020). Wilderness Medical Society Clinical Practice Guidelines for the Management of Exercise-Associated Hyponatremia: 2019 Update. Wilderness Environ. Med..

[64] McGreal K., Budhiraja P., Jain N., Yu A.S. (2016). Current challenges in the evaluation and management of hyponatremia. Kidney Dis..

[65] Hew-Butler T., Almond C., Ayus J.C., Dugas J., Meeuwisse W., Noakes T., Weschler L. (2005). Consensus Statement of the 1st International Exercise-Associated Hyponatremia Consensus Development Conference, Cape Town, South Africa 2005. Clin. J. Sport Med..

[66] Hew-Butler T. (2019). Exercise-Associated Hyponatremia. Front. Horm. Res..

[67] Rüst C.A. (2012). Higher prevalence of exercise-associated hyponatremia in triple iron ultra-triathletes than reported for ironman triathletes. Chin. J. Physiol..

[68] Sharwood K.A., Collins M., Goedecke J.H., Wilson G., Noakes T.D. (2004). Weight changes, medical complications, and performance during an Ironman triathlon. Br. J. Sports Med..

[69] Speedy D.B., Faris J.G., Hamlin M., Gallagher P.G., Campbell R.G. (1997). Hyponatremia and weight changes in an ultradistance triathlon. Clin. J. Sport Med..

[70] Nikolaidis P.T., Veniamakis E., Rosemann T., Knechtle B. (2018). Nutrition in Ultra-Endurance. State of the Art. Nutrients.

[71] Schwartz W.B., Bennett W., Curelop S., Bartter F.C. (1957). A Syndrome of Renal Sodium Loss and Hyponatremia Probably Resulting from Inappropriate Secretion of Antidiuretic Hormone. Am. J. Med..

[72] Buck E., Miles R., Schroeder J.D. (2021). Exercise-Associated Hyponatremia.

[73] Rosner M.H. (2008). Exercise-associated hyponatremia. Physician Sportsmed..

[74] Twerenbold R., Knechtle B., Kakebeeke T.H., Eser P., Müller G., Von Arx P., Knecht H., Rehrer N., Speedy D. (2003). Effects of different sodium concentrations in replacement fluids during prolonged exercise in women. Br. J. Sports Med..

[75] Oh R.C., Galer M., Bursey M.M. (2018). Found in the Field—A Soldier with Heat Stroke, Exercise-Associated Hyponatremia, and Kidney Injury. Curr. Sports Med. Rep..

[76] Holtzhausen L.M., Noakes T.D. (1997). Collapsed ultraendurance athlete: Proposed mechanisms and an approachto management. Clin. J. Sport Med..

[77] Hew-Butler T., Rosner M.H., Fowkes-Godek S., Dugas J.P., Hoffman M., Lewis D.P., Maughan R.J., Miller K.C., Montain S.J., Rehrer N.J. (2015). Statement of the Third International Exercise-Associated Hyponatremia Consensus Development Conference, Carlsbad, California, 2015. Clin. J. Sport Med..

[78] Bailey E. (2017). Electrolytes: Performance Perks and Real Food Sources NASM.org. https://blog.nasm.org/fitness/electrolytes-performance-perks-and-real-food-sources.

[79] Takamata A., Mack G.W., Stachenfeld N.S., Nadel E.R. (1995). Body temperature modification of osmotically induced vasopressin secretion and thirst in humans. Am. J. Physiol..

[80] Hew T.D., Chorley J.N., Cianca J.C., Divine J.G. (2003). The Incidence, Risk Factors, and Clinical Manifestations of Hyponatremia in Marathon Runners. Clin. J. Sport Med..

[81] Spano S.J., Reagle Z., Evans T. (2014). Symptomatic Hypotonic Hyponatremia Presenting at High Altitude. Wilderness Environ. Med..

[82] Hsieh M., Roth R., Davis D.L., Larrabe E.H., Callaway C.W. (2002). Hyponatremia in runners requiring on-sitemedical treatment at a single marathon. Med. Sci. Sport Exerc..

[83] Almond C.S., Shin A.Y., Fortescue E.B., Mannix R.C., Wypij D., Binstadt B.A., Duncan C.N., Olson D.P., Salerno A.E., Newburger J.W. (2005). Hyponatremia among runners in the boston marathon. N. Engl. J. Med..

[84] Chorley J., Cianca J., Divine J. (2007). Risk Factors for Exercise-Associated Hyponatremia in Non-Elite Marathon Runners. Clin. J. Sport Med..

[85] Mettler S., Rusch C., Frey W.O., Bestmann L., Wenk C., Colombani P.C. (2008). Hyponatremia among runners inthe zurich marathon. Clin. J. Sport Med..

[86] Siegel A.J., D’Hemecourt P., Adner M.M., Shirey T., Brown J.L., Lewandrowski K.B. (2009). Exertional dysnatremiain collapsed marathon runners: A critical role for point-of-care testing to guide appropriate therapy. Am. J. Clin. Pathol..

[87] Kipps C., Sharma S., Pedoe D.T. (2009). The incidence of exercise-associated hyponatraemia in the London marathon. Br. J. Sports Med..

[88] Lee J.K., Nio A.Q., Ang W.H. (2011). First reported cases of exercise-associated hyponatremia in Asia. Int. J. Sports Med..

[89] Hoffman M.D., Hew-Butler T., Stuempfle K.J. (2013). Exercise-associated hyponatremia and hydration status in 161-km ultramarathoners. Med. Sci. Sports Exerc..

[90] Stuempfle K.J., Lehmann D.R., Case H.S., Bailey S., Hughes S.L., McKenzie J., Evans D. (2002). Hyponatremia in a cold weather ultraendurance race. Alsk. Med..

[91] Lebus D.K., Casazza G.A., Hoffman M.D., Van Loan M.D. (2010). Can Changes in Body Mass and Total Body Water Accurately Predict Hyponatremia After a 161-km Running Race?. Clin. J. Sport Med..

[92] Hew-Butler T. (2010). Arginine Vasopressin, Fluid Balance and Exercise. Sports Med..

[93] Harris G., Reid S., Sikaris K., McCrory P. (2012). Hyponatremia is associated with higher nt-probnp thannormonatremia after prolonged exercise. Clin. J. Sport Med..

[94] Speedy D.B., Noakes T.D., Rogers I.R., Thompson J.M., Campbell R.G., Kuttner J.A., Boswell D.R., Wright S., Hamlin M. (1999). Hyponatremia in ultradistance triathletes. Med. Sci. Sports Exerc..

[95] Wharam P.C., Speedy D.B., Noakes T.D., Thompson J.M., Reid S.A., Holtzhausen L.-M. (2006). NSAID use increases the risk of developing hyponatremia during an Ironman triathlon. Med. Sci. Sports Exerc..

[96] Urso C., Brucculeri S., Caimi G. (2014). Physiopathological, epidemiological, clinical and therapeutic aspects of exercise-associated hyponatremia. J. Clin. Med..

[97] Rosner M.H. (2015). Preventing Deaths Due to Exercise-Associated Hyponatremia: The 2015 Consensus Guidelines. Clin. J. Sport Med..

[98] Bridges E., Altherwi T., Correa J.A., Hew-Butler T. (2020). Oral Hypertonic Saline Is Effective in Reversing Acute Mild-to-Moderate Symptomatic Exercise-Associated Hyponatremia. Clin. J. Sport Med..

[99] Hew-Butler T., Sharwood K., Boulter J., Collins M., Tucker R., Dugas J., Noakes T. (2007). Dysnatremia Predicts a Delayed Recovery in Collapsed Ultramarathon Runners. Clin. J. Sport Med..

[100] Spasovski G., Vanholder R., Allolio B., Annane D., Ball S., Bichet D., Decaux S., Fenske W., Hoorn E.J., Ichai C. (2014). Clinical practice guideline on diagnosis and treatment of hyponatraemia. Eur. J. Endocrinol..

[101] Rogers I.R., Hook G., Stuempfle K.J., Hoffman M.D., Hew-Butler T. (2011). An intervention study of oralversus intravenous hypertonic saline administration in ultramarathon runners with exercise-associatedhyponatremia: A preliminary randomized trial. Clin. J. Sport Med..

[102] Sanders B., Noakes T.D., Dennis S.C. (2001). Sodium replacement and fluid shifts during prolonged exercise in humans. Eur. J. Appl. Physiol..

[103] Coso J.D., Estevez E., Baquero R.A., Mora-Rodriguez R. (2008). Anaerobic performance when rehydrating with water or commercially available sports drinks during prolonged exercise in the heat. Appl. Physiol. Nutr. Metab..

[104] Domínguez R., Mata-Ordoñez F., Sánchez-Oliver A.J. (2017). Nutrición Deportiva Aplicada: Guía para Optimizar el Rendimiento.

[105] Zoorob R., Parrish M.-E.E., O’Hara H., Kalliny M. (2013). Sports Nutrition Needs. Primary Care. Clin. Off. Pract..

[106] Convertino V.A., Armstong L.E., Coyle E.F., Mack G.W., Sawka M.N., Senay L.C., Sherman W.M. (1996). American College of Sports Medicine position stand. Exercise and fluid replacement. Med. Sci. Sports Exerc..

[107] Baker L.B., Jeukendrup A.E. (2014). Optimal Composition of Fluid-Replacement Beverages. Compr. Physiol..

[108] Kenefick R.W., Cheuvront S.N. (2012). Hydration for recreational sport and physical activity. Nutr. Rev..

[109] Parsons K. (2014). The Effects of Hot, Moderate and Cold Environments on Human Health, Comfort and Performance, Human Thermal Environments.

[110] Baker L.B., Barnes K.A., Anderson M.L., Passe D.H., Stofan J.R. (2016). Normative data for regional sweat sodiumconcentration and whole-body sweating rate in athletes. J. Sports Sci..

[111] González-Alonso J., Calbet J.A.L., Nielsen B. (1998). Muscle blood flow is reduced with dehydration during prolonged exercise in humans. J. Physiol..

[112] Burke L.M. (2019). Hydration in Sport and Exercise. Heat Stress in Sport and Exercise.

[113] Cheuvront S., Carter R., Sawka M.N. (2003). Fluid balance and endurance exercise performance. Curr. Sports Med. Rep..

[114] Maughan R.J., Noakes T.D. (1991). Fluid replacement and exercise stress. A brief review of studies on fluid replacement and some guidelines for the athlete. Sports Med..

[115] Van Rosendal S.P., Coombes J.S. (2012). Glycerol Use in Hyperhydration and Rehydration: Scientific Update. Med. Sport Sci..

[116] Cairns R.S., Hew-Butler T. (2015). Incidence of Exercise-Associated Hyponatremia and Its Association with Nonosmotic Stimuli of Arginine Vasopressin in the GNW100s Ultra-endurance Marathon. Clin. J. Sport Med..

[117] Thomas D.T., Erdman K.A., Burke L.M. (2016). American College of Sports Medicine Joint Position Statement. Nutrition and Athletic Performance. Med. Sci. Sports Exerc..

[118] Sawka M.N., Burke L.M., Eichner E.R., Maughan R.J., Montain S.J., Stachenfeld N.S. (2007). American College of Sports Medicine Position Stand. Exercise and Fluid Replacement. Med. Sci. Sports Exerc..

[119] Rodriguez N.R., di Marco N.M., Langley S., American Dietetic Association, Dietitians of Canada, American College of Sports Medicine (2009). American College of Sports Medicine position stand. Nutrition and Athletic Performance. Med. Sci. Sports Exerc..

[120] Kerksick C.M., Wilborn C.D., Roberts M.D., Smith-Ryan A., Kleiner S.M., Jäger R., Collins R., Cooke M., Davis J.N., Galvan E. (2018). ISSN exercise & sports nutrition review update: Research & recommendations. J. Int. Soc. Sports Nutr..

[121] Tiller N.B., Roberts J.D., Beasley L., Chapman S., Pinto J.M., Smith L., Wiffin M., Russell M., Sparks S.A., Duckworth L. (2019). International Society of Sports Nutrition Position Stand: Nutritional considerations for single-stage ultra-marathon training and racing. J. Int. Soc. Sports Nutr..

[122] Rodriguez N.R., DiMarco N.M., Langley S. (2009). Position of the American Dietetic Association, Dietitians of Canada, and the American College of Sports Medicine: Nutrition and athletic performance. J. Acad. Nutr. Diet..

[123] Racinais S., Alonso J.M., Coutts A.J., Flouris A.D., Girard O., González-Alonso J., Périard J.D. (2015). Consensus recommendations on training and competing in the heat. Br. J. Sports Med..

[124] Shirreffs S.M., Taylor A.J., Leiper J.B., Maughan R.J. (1996). Post-exercise rehydration in man: Effects of volume consumed and drink sodium content. Med. Sci. Sports Exerc..

[125] Grozenski A., Kiel J. (2020). Basic Nutrition for Sports Participation, Part 1: Diet Composition, Macronutrients, and Hydration. Curr. Sports Med. Rep..

[126] Jeukendrup A.E., Currell K., Clarke J., Cole J., Blannin A.K. (2009). Effect of beverage glucose and sodium content on fluid delivery. Nutr. Metab..

[127] Ranchordas M.K., Tiller N.B., Ramchandani G., Jutley R., Blow A., Tye J., Drury B. (2017). Normative data on regional sweat-sodium concentrations of professional male team-sport athletes. J. Int. Soc. Sports Nutr..

[128] Associated Press (2007). Woman Dies after Water Drinking Contest. NBCnews.com. http://www.nbcnews.com/id/16614865/ns/us_news-life/t/woman-dies-after-water-drinking-contest.

[129] Hoffman M.D., Myers T.M. (2015). Case Study: Symptomatic Exercise-Associated Hyponatremia in an Endurance Runner Despite Sodium Supplementation. Int. J. Sport Nutr. Exerc. Metab..

[130] Hew-Butler T., Loi V., Pani A., Rosner M.H. (2017). Exercise-Associated Hyponatremia: 2017 Update. Front. Med..

